# Good Rehabilitation Outcomes and Improved Nutritional Status After Treatment With the Japanese Herbal Medicine Ninjin'yoeito in an Elderly Patient With Hip Fracture and Sarcopenia: A Case Report

**DOI:** 10.3389/fnut.2020.00085

**Published:** 2020-06-26

**Authors:** Akinori Morinaga, Hiroki Nakamura, Kenji Hattanmaru, Natasya Trivena Rokot, Yoko Kimura, Takashi Ito

**Affiliations:** ^1^Institute of Oriental Medicine, Tokyo Women's Medical University, Tokyo, Japan; ^2^Department of Rehabilitation, Hattanmaru Rehabilitation Hospital, Kagoshima, Japan; ^3^Pharmacological Department of Herbal Medicine, Kagoshima University Graduate School of Medical and Dental Sciences, Kagoshima, Japan

**Keywords:** ninjin'yoeito, sarcopenia, frailty, rehabilitation, body composition, hip fracture, malnutrition

## Abstract

We report a case involving a 92-year-old man who successfully received treatment with ninjin'yoeito, a Japanese herbal medicine, during the rehabilitation phase after hip fracture surgery. The patient was diagnosed with a left femoral neck fracture and underwent surgery. Two weeks after surgery, he was admitted to a rehabilitation hospital. At that time, his height, weight, body fat percentage, muscle mass, and Functional Independence Measure (FIM) score were 167 cm, 61 kg, 34.1%, 38.2 kg, and 49, respectively. For 1 month after surgery (i.e., 2 weeks after admission to the rehabilitation facility), he received rikkunshito, a traditional Japanese herbal medicine also known as Kampo medicine, for appetite loss and underwent rehabilitation. However, his appetite loss showed no improvement, and rikkunshito (7.5 g/d) was replaced with ninjin'yoeito (7.5 g/d). Two months later, although the patient's body weight and body fat percentage decreased to 56.5 kg and 21.1%, respectively, his muscle mass increased to 38.9 kg. Nutritional status evaluation indicated an improvement in the level of proteins such as transferrin, prealbumin, and retinol-binding protein, which reflected an increase in food intake. The FIM score improved from 49 to 105. No side effects were observed. The findings from this case suggest that ninjin'yoeito, which includes *Astragalus* root and *Schisandra* fruit, may be an effective treatment option for sarcopenia or frailty with appetite loss and impaired activities of daily living in aged patients.

## Introduction

Japan is becoming a super-aged society, and this trend has been accompanied by a substantial increase in the incidence of age-related disease in recent decades. Frailty due to sarcopenia associated with aging and underlying diseases has received particular attention because it impairs the quality of life (QOL) of the elderly and shortens the healthy life span. Fracture resulting from sarcopenia or frailty is one of the main reasons why elderly individuals become bedridden, which markedly impairs their QOL. Thus, rehabilitation after treatment of the fracture is important. However, many elderly individuals with fractures exhibit a state of malnutrition, which affects the outcome of rehabilitation (1–3). Although it has been shown that nutritional management is an important part of rehabilitation, some patients exhibit appetite loss, and decreased food intake and show no progress during rehabilitation.

Mosapride and acotiamide, which are indicated for the treatment of functional dyspepsia (4), are generally used to treat appetite loss. Japanese traditional herbal medicines (Kampo medicine) are also used as appetite stimulants when pharmacological treatment is ineffective in Japan. Kampo medicine, which is derived from Chinese medicine and developed independently (5), has been integrated into modern medicine owing to its high quality and safety (6). The choice of treatment is guided according to the patient's constitution and symptoms (7). Ninjin'yoeito is used for treating serious illnesses and has been approved for use in the treatment of systemic malaise, appetite loss, and anemia by the Japanese Ministry of Health, Labor and Welfare. Ninjin'yoeito is a multicomponent formulation comprising 12 crude drugs: ginseng 3 g, *Astragalus* root 1.5 g, *Atractylodes* rhizome 4 g, *Poria* sclerotium 4 g, Japanese angelica root 4 g, *Rehmannia* root 4 g, cinnamon bark 2.5 g, peony root 2 g, *Citrus unshiu* peel 2 g, polygala root 2 g, *Schisandra* fruit 1 g, and *Glycyrrhiza* 1 g. However, the effects of ninjin'yoeito on nutritional status and rehabilitation outcomes have not been reported to date.

Herein, we report a case involving an elderly man with appetite loss and decreased motivation levels during the rehabilitation phase after surgery for a femoral neck fracture. Although his rehabilitation did not progress well initially, the outcomes improved with the introduction of ninjin'yoeito as part of the treatment. Specifically, he exhibited an improvement in nutritional status and activities of daily living (ADLs), with a notable increase in lean body mass.

## Case Presentation

A 92-year-old man who experienced a fall while walking presented at an acute care hospital with left hip pain. The patient's medical history included hypertension, dyslipidemia, and a previous myocardial infarction. The patient's current medications included amlodipine besylate, pravastatin sodium, ethyl icosapentate, aspirin, and nicorandil. Prior to his falling, he was independent with his ADLs and had a good appetite. Examination revealed a left femoral neck fracture, and the patient underwent surgery as a result. Although rehabilitation was initiated immediately after surgery, the patient's motivation level was low, because of which his rehabilitation did not progress well. Moreover, he did not eat sufficiently because of appetite loss. Accordingly, treatment was initiated with rikkunshito (7.5 g/d; Tsumura & Co., Tokyo, Japan), a Japanese herbal medicine used for appetite loss and functional dyspepsia. Two weeks after surgery, the patient was transferred to our rehabilitation hospital. At that time, his height, weight, body fat percentage, muscle mass, and Functional Independence Measure (FIM) score were 167 cm, 61 kg, 34.1%, 38.2 kg, and 49, respectively. His general condition and nutritional status from admission to discharge are described in Table 1, whereas findings of body composition assessments and FIM scores during that period are described in Tables 2, 3, respectively. InBody S10 (InBody, Tokyo, Japan) was used to measure the body composition. The patient's hand grip (HG) strength and skeletal muscle mass index (SMI) were 19.6 kg and 6.42 kg/m^2^, respectively. He was diagnosed with sarcopenia according to the Asian Working Group for Sarcopenia criteria as follows: HG strength of <26 kg and SMI of <7.0 kg/m^2^ (8). He also exhibited weakness, poor endurance, and low activity, thus meeting the criteria for frailty (9).

Table 1Findings and laboratory data from admission to discharge for an elderly patient who received ninjin'yoeito, a Japanese herbal medicine, during the rehabilitation phase after hip fracture surgery.

**Table:** 

	**At admission**	**At discharge**
Height, cm	167	167
BW, kg	61.0	56.5
BMI, kg/m2	21.9	20.3
HS, kg (right)	19.6	22.6
HS, kg (left)	23.3	20.7

**Table:** 

**Nutritional status**	**At admission**	**Ninjin'yoeito treatment initiation**	**1 month after treatment initiation**	**2 months after treatment initiation**
TP, g/dL	6.4	6.8	6.7	7.1
ALB, g/dL	3.0	3.5	3.3	3.5
Tf, mg/dL		227	213	238
PreALB, mg/dL		24.7	21.9	27.9
RBP, mg/dL		2.9	2.6	3.6

*BW, body weight; BMI, body mass index; HS, handgrip strength; TP, total protein; ALB, albumin; Tf, transferrin; PreALB, prealbumin; RBP, retinol-binding protein*.

**Table 2:** Body composition of an elderly patient who received ninjin'yoeito, a Japanese herbal medicine, during the rehabilitation phase after hip fracture surgery.

	**At admission**	**1 month after admission**	**2 months after admission**	**At discharge after 2.5 months**
BW, kg	61.0	59.1	57.5	56.5
Lean mass, kg	38.2	37.5	42.0	42.2
Fat mass, kg	20.8	19.6	13.1	11.9
PBF, %	34.1	33.2	22.7	21.1
**LEAN MASS**				
Right arm, kg	2.22	2.04	2.38	2.45
Left arm, kg	2.14	2.02	2.33	2.46
Trunk, kg	19.3	18.5	20.1	20.6
Right leg, kg	6.64	6.82	6.71	6.62
Left leg, kg	6.90	6.93	6.98	6.78
SMI, kg/m^2^	6.42	6.39	6.60	6.57
BME, kcal	1,239	1,223	1,330	1,333

*PBF, percent body fat; SMI, skeletal muscle mass index; BME, basal metabolic expenditure*.

**Table 3:** Functional Independence Measure scores at admission and discharge for an elderly patient who received ninjin'yoeito, a Japanese herbal medicine, during the rehabilitation phase after hip fracture surgery.

**FIM item**	**At admission**	**At discharge**	**Maximum**	**Improvement**
Self-care	18	38	42	20
Sphincter control	2	14	14	12
Transfer	9	18	21	9
Locomotion	2	10	14	8
FIM-M total	31	80	91	49
Communication	9	13	14	4
Social cognition	9	12	21	3
FIM-C total	18	25	35	7
Total	49	105	126	56

*FIM, functional independence measure; FIM-M, FIM motor subscores; FIM-C, FIM cognitive subscores*.

Prior to the fracture, the patient was ambulatory; however, when he was transferred to our rehabilitation hospital, he initially used a wheelchair. In addition, his total FIM score was 49 (Table 3); he showed independence only while eating meals and required assistance with all other activities such as dressing, defecating, and moving around. Furthermore, he felt like eating only occasionally and ingested a variable amount of food, with an average daily calorie intake of 992 kcal. Although conventional rehabilitation (joint range-of-motion training and ADL training) was ongoing, he typically remained in bed. Considering the constant appetite loss and decreased motivation levels, rikkunshito treatment was replaced with ninjin'yoeito treatment (7.5 g/d; Kracie Pharma, Ltd., Tokyo, Japan) twice a day before meals according to the package insert from day 15. There was no restriction on other drugs during the ninjin'yoeito treatment. Subsequently, his food intake gradually stabilized, and the average daily calorie intake during the period of ninjin'yoeito treatment increased to 1,159 kcal. In particular, the average daily calorie intake increased to 1,234 kcal during the last month of hospitalization. Moreover, there was an increase in the serum levels of transferrin, prealbumin, and retinol-binding protein, which are typical indices of nutritional status. The duration of ninjin'yoeito treatment was 2 months. Although the patient's HG strength and SMI still indicated sarcopenia, he became almost fully independent with his lifestyle activities and exhibited a total FIM score of 105, with markedly improved motor subscores, at the time of discharge 2.5 months after admission. Despite weight loss and decreased body fat percentage relative to the time of admission, there was an increase in lean body mass. During the period of hospitalization, the patient had good adherence and reported no adverse events related to the ninjin'yoeito treatment.

The treatment protocol for this case was approved by the ethics committee of Hattanmaru Rehabilitation Hospital, and the patient provided written informed consent for publication of this report.

## Discussion

In this report, we describe the successful rehabilitation after surgery for a femoral neck fracture in an elderly patient who was associated with improvement of average daily calorie intake from 992 to 1,159 kcal, muscle mass from 38.2 to 38.9 kg, and FIM score from 49 to 105. Because of appetite loss and decreased motivation levels, the patient's rehabilitation did not progress well. Even after 2-week administration of rikkunshito, used for the management of appetite loss and functional dyspepsia in Japan (10), there was no improvement in the amount of food ingested by the patient. Therefore, his treatment was switched to ninjin'yoeito, which is used to manage appetite loss, sarcopenia, mild depression, and fatigue during cancer treatment (11). The initiation of ninjin'yoeito treatment increased the patient's daily calorie intake, thus resulting in good progress of rehabilitation and an improvement in ADLs. In addition, there was an increase in the serum levels of transferrin, prealbumin, and retinol-binding protein, which are typical indices of nutritional status. Ohsawa et al. (12) reported that ninjin'yoeito maintained the skeletal muscle mass through the improvement of amino acid metabolism and might improve the capacity of amino acid storage in the skeletal muscle in tumor-bearing mice. In the present case, muscle mass increased from 38.2 to 38.9 kg with ninjin'yoeito treatment. However, with no cancer-induced sarcopenia, ninjin'yoeito might improve the amino acid metabolism and storage in the skeletal muscle. Whereas the body fat percentage decreased (it exceeded 30% at the time of admission), the trunk and appendicular skeletal muscle mass increased. Malafarina and colleagues (13) evaluated elderly patients with hip fracture and found that intake of oral nutritional supplements in addition to a standard diet helped in maintaining body weight, fat mass, and muscle mass, all of which exhibited a decrease in the control group. The increase in skeletal muscle mass and decrease in body weight and fat mass in the present patient can be attributed to improved ADLs and increased calorie consumption.

To date, clinical studies on the use of ninjin'yoeito have demonstrated efficacy in patients with lung cancer (11), pulmonary non-tuberculous mycobacteriosis (14), pharyngeal vascular malformation (15), anemia associated with chronic hepatitis C (16), chronic inflammation and poor QOL due to hemodialysis (17), depression associated with Alzheimer-type dementia (when used in combination with donepezil) (18), and frailty (19–22). Rikkunshito is a formulation comprising eight crude drugs, five of which are also included in ninjin'yoeito: ginseng, *Atractylodes* rhizome, *Poria* sclerotium, *C. unshiu* peel, and *Glycyrrhiza*. Rikkunshito has been reported to enhance ghrelin signaling and consequently improve appetite loss (23). Hesperidin, a component of *C. unshiu* peel, has been reported to inhibit the binding of serotonin to serotonin receptors, thereby increasing the secretion of ghrelin, an orexigenic hormone, from the stomach (23). Goswami et al. (24) reported that ninjin'yoeito activates the orexigenic peptide neuropeptide Y (NPY) in the arcuate nucleus of the hypothalamus, independent of ghrelin, and has a therapeutic potential to improve appetite in the elderly patients with ghrelin resistance. This suggests a difference in efficacy between rikkunshito and ninjin'yoeito with regard to appetite stimulation. *Astragalus* root, which is not included in rikkunshito but is a constituent of ninjin'yoeito, has been reported to improve cancer-associated anorexia in patients with advanced cancer (25). In addition, ogikenchuto, a Kampo formula that includes *Astragalus* root, has been reported to improve ADLs in bedridden elderly patients in a Japanese study (26). Conversely, there are no similar published reports regarding rikkunshito. Kawamata et al. (26) reported that ogikenchuto improved motivation levels for rehabilitation and ADLs in two elderly bedridden patients, one of which received rikkunshito before ogikenchuto and showed a poor response. In the present case, the beneficial effects of ninjin'yoeito on appetite loss and decreased ADLs could be attributed to *Astragalus* root, which is not present in rikkunshito. *Schisandra* fruit, another ingredient of ninjin'yoeito, has also been reported to enhance exercise-induced adaptive muscle strengthening in aged mice (27) and improve endurance and metabolism in the skeletal muscle of exercised rats (28). Thus, it may be responsible for the improvement in ADLs and increase in muscle mass in our patient.

## Conclusion

We described herein the successful use of ninjin'yoeito for the management of appetite loss, malnutrition, and decreased ADLs during the rehabilitation phase after surgery for femoral neck fracture in an elderly patient. Our findings suggest that Kampo medicine containing *Astragalus* root and *Schisandra* fruit, such as ninjin'yoeito, may be an effective treatment option for sarcopenia or frailty with malnutrition due to appetite loss. Furthermore, ninjin'yoeito may be applied to other types of fracture due to sarcopenia. Further large clinical trials on the efficacy of ninjin'yoeito treatment for sarcopenia or frailty are warranted.

## Data Availability Statement

The datasets generated for this study are available on request to the corresponding author.

## Ethics Statement

The studies involving human participants were reviewed and approved by the ethics committee of Hattanmaru Rehabilitation Hospital. The patients/participants provided their written informed consent to participate in this study.

## Author Contributions

AM designed the study and wrote the initial draft of the manuscript. All other authors have contributed to data collection and interpretation and critically reviewed the manuscript. All authors approved the final version of the manuscript and agree to be accountable for all aspects of the work, while ensuring that questions related to the accuracy or integrity of any part of the work are appropriately investigated and resolved.

## Conflict of Interest

The authors declare that the research was conducted in the absence of any commercial or financial relationships that could be construed as a potential conflict of interest.

## Abbreviations

FIM: Functional Independence Measure
QOL: quality of life
ADLs: activities of daily living
HG: hand grip
SMI: skeletal muscle mass index
NPY: neuropeptide Y.

## References

[1] Wakabayashi H Sashika H Malnutrition is associated with poor rehabilitation outcome in elderly inpatients with hospital-associated deconditioning a prospective cohort study. J Rehabil Med. (2014) 46:277–82. 10.2340/16501977-1258 24213734

[2] Niitsu M Ichinose D Hirooka T Mitsutomi K Morimoto Y Sarukawa J. Effects of combination of whey protein intake and rehabilitation on muscle strength and daily movements in patients with hip fracture in the early postoperative period. Clin Nutr. (2016) 35:943–9. 10.1016/j.clnu.2015.07.006 26216195

[3] Nishioka S Wakabayashi H Momosaki R. Nutritional status changes and activities of daily living after hip fracture in convalescent rehabilitation units: a retrospective observational cohort study from the Japan rehabilitation nutrition database. J Acad Nutr Diet. (2018) 118:1270–6. 10.1016/j.jand.2018.02.012 29752190

[4] Miwa H Kusano M Arisawa T Oshima T Kato M Joh T. Evidence-based clinical practice guidelines for functional dyspepsia. J Gastroenterol. (2015) 50:125–39. 10.1007/s00535-014-1022-3 25586651

[5] Kuchta K Traditional Japanese kampo medicine - History of ideas and practice; Part 1: from ancient shamanic practice to the medical academies of Edo. Tradit Kampo Med. (2019) 6:49–56. 10.1002/tkm2.1209

[6] Motoo Y Seki T Tsutani K. Traditional Japanese medicine, Kampo: its history and current status. Chin J Integr Med. (2011) 17:85–7. 10.1007/s11655-011-0653-y 21390572

[7] Satoh H. Kampo pharmacology: integration and fusion between Kampo medicine and Western Medicine–the efficacy of Kampo medicine. Nihon Yakurigaku Zasshi. (2014) 143:56–60. 24531896

[8] Chen LK Liu LK Woo J Assantachai P Auyeung TW Bahyah KS. Sarcopenia in Asia: consensus report of the Asian Working Group for Sarcopenia. J Am Med Dir Assoc. (2014) 15:95–101. 10.1016/j.jamda.2013.11.025 24461239

[9] Fried LP Tangen CM Walston J Newman AB Hirsch C Gottdiener J. Frailty in older adults: evidence for a phenotype. J Gerontol A Biol Sci Med Sci. (2001) 56:M146–56. 10.1093/gerona/56.3.M146 11253156

[10] Tominaga K Sakata Y Kusunoki H Odaka T Sakurai K Kawamura O. Rikkunshito simultaneously improves dyspepsia correlated with anxiety in patients with functional dyspepsia: a randomized clinical trial (the DREAM study). Neurogastroenterol Motil. (2018) 30:e13319. 10.1111/nmo.13319 29498457

[11] Kamei T Kumano H Iwata K Nariai Y Matsumoto T. The effect of a traditional Chinese prescription for a case of lung carcinoma. J Altern Complement Med. (2000) 6:557–9. 10.1089/acm.2000.6.557 11152062

[12] Ohsawa M Maruoka J Inami C Iwaki A Murakami T Ishikura KI. Effect of ninjin'yoeito on the loss of skeletal muscle function in cancer-bearing mice. Front Pharmacol. (2018) 9:1400. 10.3389/fphar.2018.01400 30555329 PMC6284053

[13] Malafarina V Uriz-Otano F Malafarina C Martinez JA Zulet MA. Effectiveness of nutritional supplementation on sarcopenia and recovery in hip fracture patients. A multi-centre randomized trial. Maturitas. (2017) 101:42–50. 10.1016/j.maturitas.2017.04.010 28539168

[14] Nogami T Sekiya N Mitsuma T Yamaguchi T. A case of pulmonary *Mycobacterium fortuitum* infection successfully treated with Kampo treatments. Kekkaku. (2006) 81:525–9. 16972656

[15] Ogawa-Ochiai K Osuga K Hidaka K Nakahata K Tazuke Y Yamamoto Y. A case of extensive pharyngeal vascular malformation successfully treated with Kampo medicine. Auris Nasus Larynx. (2018) 45:190–3. 10.1016/j.anl.2017.03.008 28389161

[16] Motoo Y Mouri H Ohtsubo K Yamaguchi Y Watanabe H Sawabu N. Herbal medicine Ninjinyoeito ameliorates ribavirin-induced anemia in chronic hepatitis C: a randomized controlled trial. World J Gastroenterol. (2005) 11:4013–7. 10.3748/wjg.v11.i26.4013 15996025 PMC4502096

[17] Hsiao PJ Lin KS Chiu CC Chen HW Huang JS Kao SY. Use of traditional Chinese medicine (Ren Shen Yang Rong Tang) against microinflammation in hemodialysis patients: an open-label trial. Complement Ther Med. (2015) 23:363–71. 10.1016/j.ctim.2015.03.002 26051571

[18] Kudoh C Arita R Honda M Kishi T Komatsu Y Asou H. Effect of ninjin'yoeito, a Kampo (traditional Japanese) medicine, on cognitive impairment and depression in patients with Alzheimer's disease: 2 years of observation. Psychogeriatrics. (2016) 16:85–92. 10.1111/psyg.12125 25918972

[19] Ohsawa M Tanaka Y Ehara Y Makita S Onaka KA Possibility of simultaneous treatment with the multicomponent drug ninjin'yoeito for anorexia apathy and cognitive dysfunction in frail Alzheimer's disease patients: an open-label pilot study. J Alzheimers Dis Rep. (2017) 1:229–35. 10.3233/ADR-170026 30480240 PMC6159634

[20] Kuniaki H Akihiko T Tetsuya H Hatsuko M Tomoko K Shin O. Improvement in frailty in a patient with severe chronic obstructive pulmonary disease after ninjin'yoeito therapy: a case report. Front Nutr. (2018) 5:71. 10.3389/fnut.2018.00071 30234120 PMC6131554

[21] Sakisaka N Mitani K Sempuku S Imai T Takemoto Y Shimomura H. A clinical study of ninjin'yoeito with regard to frailty. Front Nutr. (2018) 5:73. 10.3389/fnut.2018.00073 30320119 PMC6165905

[22] Kimura Y Ito T Ninjin'yoeito for symptoms of frailty: successful treatment of three cases. Tradit Kampo Med. (2019) 6:37–40. 10.1002/tkm2.1206

[23] Suzuki H Asakawa A Amitani H Fujitsuka N Nakamura N Inui A. Cancer cachexia pathophysiology and translational aspect of herbal medicine. Jpn J Clin Oncol. (2013) 43:695–705. 10.1093/jjco/hyt075 23737606

[24] Goswami C Dezaki K Wang L Inui A Seino Y Yada T. Ninjin-yoeito activates ghrelin-responsive and unresponsive NPY neurons in the arcuate nucleus and counteracts cisplatin-induced anorexia. Neuropeptides. (2019) 75:58–64. 10.1016/j.npep.2019.03.001 30948035

[25] Lee JJ Lee JJ. A phase II study of an herbal decoction that includes *Astragali radix* for cancer-associated anorexia in patients with advanced cancer. Integr Cancer Ther. (2010) 9:24–31. 10.1177/1534735409359180 20150220

[26] Kawamata H Tosa H Terasawa K Two cases of report of bedridden aged patients effectively treated with a kampo prescription ogi-kenchu-to. Kampo Med. (1996) 47:253–60. 10.3937/kampomed.47.253

[27] Kim KY Ku SK Lee KW Song CH An WG. Muscle-protective effects of *Schisandrae* Fructus extracts in old mice after chronic forced exercise. J Ethnopharmacol. (2018) 212:175–87. 10.1016/j.jep.2017.10.022 29107647

[28] Kim YJ Yoo SR Chae CK Jung UJ Choi MS. Omija fruit extract improves endurance and energy metabolism by upregulating PGC-1α expression in the skeletal muscle of exercised rats. J Med Food. (2014) 17:28–35. 10.1089/jmf.2013.3071 24456352

